# Targeting Muscle-Resident Single Cells Through *in vivo* Electro-Enhanced Plasmid Transfer in Healthy and Compromised Skeletal Muscle

**DOI:** 10.3389/fphys.2022.834705

**Published:** 2022-04-01

**Authors:** Francesca Florio, Silvia Accordini, Michela Libergoli, Stefano Biressi

**Affiliations:** ^1^Department of Cellular, Computational and Integrative Biology (CIBIO), University of Trento, Trento, Italy; ^2^Dulbecco Telethon Institute at University of Trento, Trento, Italy

**Keywords:** muscle stem cells, endothelial cells, fibroadipogenic progenitors, electroporation, collagen, muscular dystrophy, aging

## Abstract

Skeletal muscle is composed of syncytial muscle fibers, and by various mononucleated cellular types, such as muscle stem cells, immune cells, interstitial and stromal progenitors. These cell populations play a crucial role during muscle regeneration, and alterations of their phenotypic properties have been associated with defective repair and fibrosis in aging and dystrophic muscle. Studies involving *in vivo* gene modulation are valuable to investigate the mechanisms underlining cell function and dysfunction in complex pathophysiological settings. Electro-enhanced transfer of plasmids using square-wave generating devices represents a cost-effective approach that is widely used to transport DNA to muscle fibers efficiently. Still, it is not clear if this method can also be applied to mononuclear cells present in muscle. We demonstrate here that it is possible to efficiently deliver DNA into different muscle–resident cell populations *in vivo*. We evaluated the efficiency of this approach not only in healthy muscle but also in muscles of aging and dystrophic animal models. As an exemplificative application of this method, we used a strategy relying on a reporter gene-based plasmid containing regulatory sequences from the *collagen 1 locus*, and we determined collagen expression in various cell types reportedly involved in the production of fibrotic tissue in the dystrophic settings. The results enclosed in this manuscript reveal the suitability in applying electro-enhanced transfer of plasmid DNA to mononucleated muscle-resident cells to get insights into the molecular events governing diseased muscle physiology.

## Introduction

The basic functional cell units of skeletal muscle are the myofibers, which consists of single elongated multinucleated cells formed during development following proliferation and fusion process of muscle progenitor cells (Biressi et al., 2007; Bryson-Richardson and Currie, 2008; Comai and Tajbakhsh, 2014). Following injury, the skeletal muscle has a remarkable capacity to regenerate and restore its physiological tissue architecture and functionality. Skeletal muscle regeneration consists of several highly coordinated cellular processes, which lead to the formation of tissue similar to the uninjured muscle (Kuang and Rudnicki, 2008; Baghdadi and Tajbakhsh, 2018). Muscle regeneration requires the orchestrated contribution of at least two different classes of cellular players in the tissue: (1) the muscle stem cells (also known as muscle satellite cells, MuSCs), which are quiescent in uninjured muscle, but become activated after an injury, proliferate and eventually fuse to differentiate into new myofibers (Scharner and Zammit, 2011); (2) support cells, which consist of a heterogeneous group of cells found in the interstitial spaces between myofibers or associated to the vasculature (Wosczyna and Rando, 2018). They include tissue-resident cells and cells infiltrating from the circulation, and the disruption of their normal contributions has been associated with incomplete regeneration. This aspect is highlighted by mesenchymal stromal cells, called fibroadipogenic progenitors (FAPs), which are required for muscle regeneration and maintenance (Joe et al., 2010; Uezumi et al., 2010; Wosczyna et al., 2019). Moreover, experiments conducted by multiple groups have identified endothelial cells, pericytes and different subpopulations of immune cells, including macrophages, eosinophils and regulatory T cells, as necessary components for proper MuSCs function and effective muscle restoration (Shen et al., 2008; Burzyn et al., 2013; Heredia et al., 2013; Kostallari et al., 2015; Lemos et al., 2015; Latroche et al., 2017; Liu et al., 2017; Verma et al., 2018).

The homeostatic importance of skeletal muscle regeneration appears clear when considering physiological aging or genetic pathologies such as muscular dystrophies. Both intrinsic and extrinsic cues negatively impact MuSCs regenerative properties in old muscles (Muñoz-Cánoves et al., 2020; Rando and Jones, 2021). MuSCs from dystrophic muscles reportedly present several abnormalities (Morgan and Cohen, 1974; Ionasescu and Ionasescu, 1982; Blau et al., 1983; Delaporte et al., 1984, 1990; Jasmin et al., 1984; Melone et al., 1999; Furling et al., 2001; Alexakis et al., 2007; Rhoads et al., 2013; Biressi et al., 2014; Dumont et al., 2015; Pessina et al., 2015). The functional interactions between MuSCs and support cells and among different subpopulations of inflammatory and stromal cells also appear to be compromised in aging and diseased muscle (Villalta et al., 2009; Mozzetta et al., 2013; Wang et al., 2015). These alterations are likely contributing to the defective regeneration and lead to the chronic accumulation of inert fibrotic tissue that is observable in aging and dystrophic muscle (Serrano and Munoz-Canoves, 2010). Little is known about the mechanisms underlining fibrosis in diseased muscle tissue, and different cellular sources have been reported to contribute to the excessive accumulation of extracellular matrix components. FAPs (or their derivatives) have been proposed as major contributors (Uezumi et al., 2011; Mueller et al., 2016). However, an increasing amount of evidence indicates that other cell types can potentially contribute to fibrosis. MuSCs have the propensity to adopt fibrogenic phenotypes in the setting of both aged and dystrophic muscle (Brack et al., 2007; Biressi et al., 2014; Pessina et al., 2015). This parallels a similar behavior documented for endothelial cells and cells belonging to the hematopoietic lineage in the dystrophic settings (Pessina et al., 2015; Wang et al., 2016).

The *in vivo* alteration of gene expression is a powerful tool to investigate muscle biology, including the properties of MuSCs and other muscle cell populations. Genetic or pharmacological down- and up-regulation of various genes have been widely exploited to get insights into the basic mechanisms controlling productive regeneration in healthy muscle and pathological defective repair. Therapeutic gene expression and nucleic acid delivery have been induced in different animal models to restore physiological gene expression and counteract the progression of various muscle genetic diseases, and recently also entered the clinical arena (Bengtsson et al., 2016; Chamberlain and Chamberlain, 2017; Schneider and Aartsma-Rus, 2021). Genetic manipulation by transient or stable transfer of nucleic acids in the tissues of live organisms has been applied to skeletal muscle by different approaches, involving both viral vectors and non-viral strategies (Braun, 2008; Hollinger and Chamberlain, 2015). Among the non-viral strategies, electro-enhanced plasmid transfer (i.e., electroporation) has gained a special position over the last three decades due to its simplicity, safety, and cost-effectiveness that perfectly suit the time and practical constraints typical of the academic research. *In vivo* electro-enhanced plasmid transfer consists of a physical method of gene delivery relying on the cellular uptake of plasmid DNA facilitated by an electric field transmitted by electrodes placed on each side of the injection site and connected to a square-wave pulse generator (Somiari et al., 2000; Bloquel et al., 2004; Sokołowska and Błachnio-Zabielska, 2019). Specialized technical skills or complex instrumentation are not needed to electroporate skeletal muscle. Therefore, it is not surprising that electro-enhanced plasmid delivery has become one of the elective methods to be used in gain- or loss-of-function studies targeting skeletal muscle, particularly at the preclinical laboratory level.

Noteworthy, the great majority of the studies in this field have been centered on the delivery of nucleic acids to multinucleated muscle fibers, and little focus has been given to the mononucleated cells that are coexisting with mature fibers in the muscle tissue. Lentiviral vectors have been reported to target muscle stem cells (Kobinger et al., 2003; Kimura et al., 2010; Jonuschies et al., 2014). Nevertheless, conflicting information is available for non-viral delivery methods, such as electroporation (Peng et al., 2005; Wong et al., 2005). Despite circumstantial evidence in immune and interstitial cells, a systematic quantification of the efficiency of electro-enhanced plasmid transfer to muscle-resident mononucleated cells that are different from MuSCs still needs to be performed (Dupuis et al., 2000; Grønevik et al., 2003; Ratanamart et al., 2010). Through a cytofluorimetric analysis based on well-established lineage markers, we demonstrate here that it is possible to target various defined mononucleated cell subtypes (i.e., FAPs, endothelial cells, cells of the hematopoietic lineage, MuSCs) that reside in skeletal muscles by using an electro-enhanced plasmid delivery protocol. Notably, we characterized the process of electro-enhanced DNA transfer to mononucleated cells in healthy muscle and muscles affected by alterations in structure and function, such as muscles of aging and dystrophic animal models. Moreover, by applying a strategy relying on the electroporation of a reporter gene-expressing plasmid, we determined the extent of collagen expression in various cell types present in fibrotic dystrophic and old muscles. The results enclosed in this manuscript reveal the suitability of electro-enhanced DNA delivery in targeting muscle-resident cells to get insights into the cellular and molecular events governing diseased muscle physiology.

## Materials and Methods

### Mice

Animals were maintained with access to food and water *ad libitum* and kept at a constant temperature on a 12:12 h light/dark cycle. *C57BL/6J* mice (The Jackson Laboratory, no. 000664) were used as *wild type* animals. B6Ros.Cg-*Dmd^mdx–*4*Cv/J^* mice (The Jackson Laboratory, no. 002378, herein referred to as *mdx^4Cv^*) were used as dystrophic mice. Animal care and experimental procedures were conducted in accordance with the Ethical Committee of the University of Trento and were approved by the Italian Ministry of Health (Authorization No. 62/2020-PR).

### *In vivo* Gene Transfer and Electroporation

Tibialis anterior (*TA*) muscles were electroporated as previously described with minor modifications (Magarò et al., 2021). Briefly, mice were anesthetized by inhaled isoflurane, the hindlimb skin was shaved and disinfected with 70% ethanol. Plasmids were injected with a 0.5 ml insulin syringe through a 27-gauge needle into *TA* muscles in a constant volume of 40 μL. Plasmid DNA was purified using endofree plasmid kits (Qiagen) and dissolved in sterile PBS at the concentration of 2 μg/μL. The following expression vectors were used: pCMV-LacZ (Clontech), pEGFP-N3 (Clontech, hereinafter referred to as GFP), ptdTomato-N1 (Clontech, hereinafter referred to as tdTomato), pOBCol2.3-GFPemd (hereinafter referred to as Col1-GFP). pOBCol2.3-GFPemd was a gift from David Rowe (Addgene plasmid # 110210^1^). For specific experiments a mix of GFP- and tdTomato- expressing plasmids (in 10:1 ratio) or a mix of Col1-GFP- and tdTomato- expressing plasmids (in 10:1 ratio) was injected. Unless otherwise stated, PBS was injected in samples used as negative controls. Platinum plated 5 mm tweezer-style electrodes (BTX) were then applied over the muscle to encompass the injection area. Electrode jelly was used on the electrode plates to ensure conduction. Current was delivered 5 min after DNA injection as a constant current, square-wave pulse with a digital Stimulator (Intracel TSS20 Ovodyne electroporator combined with Intracel EP21 current amplifier). The characteristics of the electric field applied were 200 V/cm, 20 ms amplitude, 1 Hz, eight consecutive pulses. Two series of pulses were applied, one on the most proximal part of the *TA* and the other on the most distal one. An analgesic was administered subcutaneously at the end of the procedure. Animals were kept warm until recovery, and then returned to their cages. When indicated, *mdx^4*Cv*^ TA* muscles were injected with 25 μl of bovine hyaluronidase (H-4272; Sigma) at the concentration of ∼0.4 U/l in saline 2 h prior to plasmid injection and electroporation, as previously described (McMahon et al., 2001; Gollins et al., 2003; Molnar et al., 2004).

### Muscle Injury

Mice were anesthetized with isoflurane, hindlimb skin was shaved, and 50 μl of cardiotoxin from *Naja pallida* snake venom (Latoxan) resuspended at the concentration of 0.1 mg/ml in PBS were injected into the mid-belly of *TA* muscles. Depending on the experiment, muscle damage was induced 1.5 days prior to electroporation or 1 week after electroporation.

### Immunofluorescence

For immunofluorescence studies, muscles were fixed for 4 h using 0.5% paraformaldehyde and transferred to 30% sucrose overnight. Muscles were frozen in optimum cutting temperature compound (OCT) (Histo-Line Laboratories). Cryosections (8 μm) were fixed with 4% paraformaldehyde for 10 min at room temperature, and processed for immunofluorescence according to standard protocols with primary antibodies (rabbit anti-GFP, Invitrogen, 1: 500; rat anti-mouse F4/80, Biolegend, 1:50) followed by incubation with Alexa Fluor 488/594 donkey secondary antibodies (Invitrogen). Incubation for 10 min with 10 μg/ml Hoechst in PBS was used to stain the nuclei. Samples were mounted using Fluroshield histology mounting medium (Sigma).

### X-Gal Staining

X-gal staining was used to detect the LacZ reporter gene expression after pCMV-LacZ plasmid injection and electroporation. Muscles were dissected 1 or 3 weeks after electroporation and fixed in 4% paraformaldehyde at 4°C for 15 min, then washed in PBS and stained at 37°C for 3-5 h in X-gal buffer [1 mg/ml 5 bromo-4-chloro-3-indolyl-β-D-galactoside, 5 mM K_4_Fe(CN)_6_, 5 mM K_3_Fe(CN)_6_, 2 mM MgCl_2_ in PBS]. When the β-galactosidase (β-gal) was expressed by the muscles, a typical blue color was observed. The X-gal buffer was removed and muscles were washed in PBS prior to images acquisition.

### Imaging

X-gal images were acquired using an optical stereomicroscope (MZ10F; Leica) and processed using LAS X software (Leica). The GFP and tdTomato intensities of dissected muscles and *in vivo* muscle were acquired using the Bruker *In Vivo* Xtreme I imaging system. Prior to the *in vivo* analysis, mice were anesthetized by inhaled isoflurane, the hindlimb skin was shaved and the animals were placed inside the instrument with the *TA* facing the camera. The GFP and tdTomato intensities were then evaluated with the Bruker MISE software as number of photons/sec/mm^2^. Immunofluorescence images were acquired using a Zeiss Axio Observer Z1 optical microscope equipped with a monochrome camera (AxioCam 503 mono D).

### Muscle Single Cells Isolation and Fluorescence-Activated Cell Sorting Analysis

Hindlimb muscles from young *C57BL/6J* (∼2 months-old), adult *C57BL/6J* (∼1 year-old), old *C57BL/6J* (∼24-26 months-old) and adult *mdx^4Cv^* (∼1 year-old) mice were dissected 1, 2 or 3 weeks after the *in vivo* gene transfer and processed as previously described to obtain mononucleated cells (Liu et al., 2015). Muscles were washed in Wash Medium (Ham’s F-10 supplemented with 10% FBS, 1% L-glutamine and 1% penicillin-streptomycin), added to Muscle Dissociation Buffer [500 U/ml collagenase II (Worthington Biochemical Corporation) prepared in Ham’s F-10], minced with scissor, incubated in a 37°C water bath with agitation (70 rpm) for 40 min. After incubation samples were centrifuged at 500g for 5 min, and the pellet was dissolved in dispase (11 U/ml, GIBCO) and collagenase II (2000 U/ml) and triturated with a 10-ml serological pipette. Samples were incubated in a 37°C water bath with agitation (70 rpm) for 20 min, then passed through 18- and 19-gauge needles by using a 10-ml syringe to allow mechanical dissociation into single cells. Samples were centrifuged at 500g for 10 min at 4°C, the pellet was resuspended and filtered through a 40-μm nylon cell strainer (Euroclone), centrifuged at 500g for 10 min at 4°C, then resuspended in Wash Medium. Cells were incubated on a rotating wheel (10 rpm) for 45 min at 4°C with primary antibodies to mark FAPs, MuSCs, hematopoietic and endothelial cells. A list of fluorescence-activated cell sorting (FACS) antibodies used is enclosed in Supplementary Table 1. Samples were washed, APC Streptavidin (1:100, BioLegend) was added and samples were incubated on a rotating wheel (10 rpm) for 20 min at 4°C, then washed and resuspended in a sorting buffer (Wash Medium + PBS with 1.5 mM EDTA, 2% BSA, 1% L-glutamine and 1% penicillin-streptomycin, 1:1 ratio). Samples were finally filtered through cell strainer cap tubes (Thermo Fisher Scientific).

FACSAria III cell sorter (BD Biosciences) was used to analyze the uptake of the electroporated plasmids in mononucleated muscle cells. Physical parameters as forward scatter (FSC) and side scatter (SSC) were used to exclude cell clumps and debris. Hematopoietic cells were identified by positive selection with anti-CD45 antibody, endothelial cells were identified by positive selection with anti-CD31 antibody or negative selection with anti-CD45 antibody followed by positive selection with anti-CD31 antibody, MuSCs were identified by negative selection with anti-CD31, anti-CD45, anti-sca1 antibodies and positive selection with anti-vcam antibody (Liu et al., 2015), FAPs were identified by negative selection with anti-CD31 and anti-CD45 antibodies and positive selection with anti-sca1 antibody (Liu et al., 2015) (Supplementary Figure 1A). Control samples (i.e., sample injected with PBS and electroporated or samples not electroporated) were used in the FACS analysis to set gates specifically for *C57BL/6J* or *mdx^4Cv^* samples to calculate the percentage of electroporated cells within each cell population. In the experiments with the Col1-GFP and tdTomato plasmids mix, a control sample was used to set the tdTomato^+ve^ (TOM^+ve^) cells gates within each cell populations. TOM^+ve^ cells (i.e., electroporated cells) were then analyzed for the GFP expression (i.e., collagen1-GFP expression). The median values of the tdTomato and GFP intensities within the cell populations were calculated using the FlowJo software. The tdTomato intensity was used as a normalizer. For specific experiments, MuSCs, FAPs, and CD31^+ve^ cells were processed after sorting for RNA extraction and real time polymerase chain reaction (RT-PCR).

### RNA Extraction and Quantitative Real-Time PCR

Total RNA was extracted from FACS isolated cells using TRIzol Reagent (Invitrogen) according to manufacturer’s instructions. RNA was quantified using NanoDrop spectrophotometer and reverse transcribed using High Capacity cDNA Reverse Transcription Kit (Thermo Fisher Scientific). Gene expression was measured by quantitative RT-PCR using SYBR Green Master Mix (Thermo Fisher Scientific) and a C1000 Touch thermocycler-CFX96 Real Time System (Biorad). Primers spanning exon-exon junctions were used (col1a1 Fw: TCC GGC TCC TGC TCC TCT TA; col1a1 Rev: GTA TGC AGC TGA CTT CAG GGA TGT). The level of each transcript was measured using mouse HPRT (hypoxanthine-guanine phosphoribosyltransferase) mRNA levels as normalizer (mHPRT Fw: AAC TGG AAA GAA TGT CTT GAT TGT; mHPRT Rev: GAA TTT CAA ATC CAA CAA AGT CTG G).

### Statistical Analysis

Unless otherwise stated, experiments presented here were repeated at least three times and quantitative data are presented as mean ± SEM. Statistical analysis was performed using GraphPad Prism 8. Unpaired Parametric Student’s *t*-test (two-tailed) was performed for comparison between two groups. Statistical significance is expressed with the p-value (p), which is reported in each graph.

## Results

### *In vivo* Electroporation of Skeletal Muscles’ Mononuclear Cells

**FIGURE 1:**
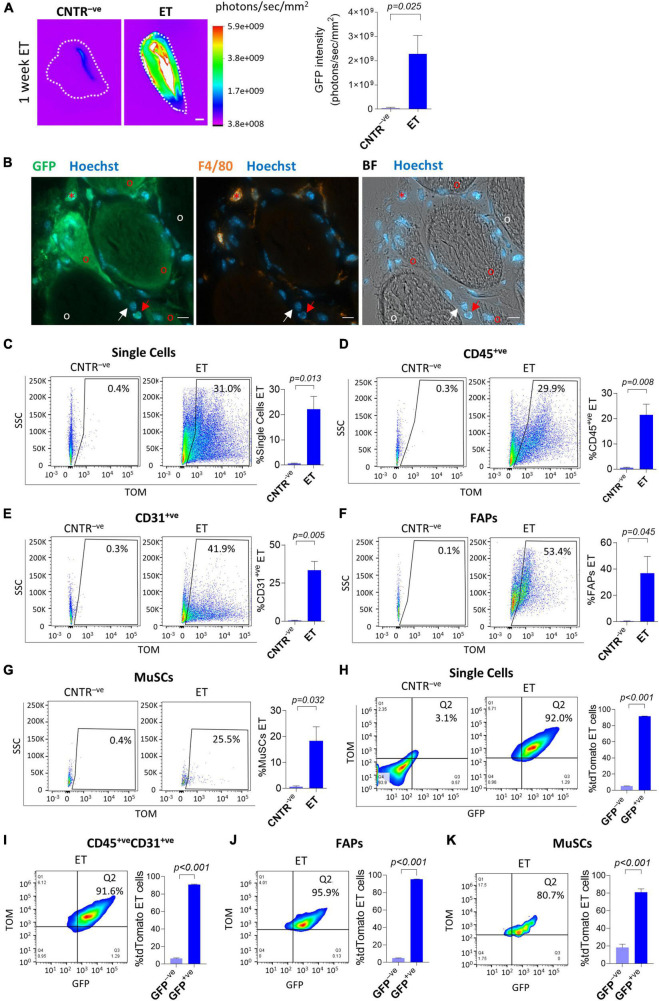
*In vivo* electroporation of whole muscles and single cells. **(A)** GFP intensity measurement (photons/sec/mm^2^) of whole *gastrocnemius* (CNTR^–^ *^ve^*) or *TA* (ET) from ∼12 months-old *C57BL/6J* mice injected with GFP plasmid and electroporated (ET) or not injected nor electroporated (CNTR^–^ *^ve^*). Scale bar: 2.5 mm. White color in the figure indicates photons/sec/mm^2^ > 5.9e+009. Refer also to Supplementary Figure 2A for the corresponding muscles reflectance images. The analysis was performed using Bruker *In Vivo* Xtreme I imaging system. Muscles were collected 1 week after electroporation and scanned *ex vivo*. *N* = 5 (CNTR^–^ *^ve^*), *N* = 6 (ET). **(B)** Representative immunofluorescence of *TA* from ∼8 months-old *C57BL/6J* mice injected with GFP plasmid and electroporated. Muscles were stained with anti-GFP (green), anti-F4/80 (orange) antibodies, and Hoechst (blue). The analysis was performed 1 week after electroporation. BF: Bright Field. Scale bar: 10 μm. Note the presence of electroporated (GFP^+ve^) F4/80^+ve^ cells (red asterisks), * and F4/80^–^ *^ve^* mononucleated muscle cells (red arrows) nearby electroporated (GFP^+ve^) muscle fibers (red circles). White arrows and circles are, respectively, marking examples of non-electroporated (GFP^–^ *^ve^*) F4/80^–^ *^ve^* mononucleated muscle cells and muscle fibers. *N* = 3. Refer to Supplementary Figure 3A for additional images collected from different mice. **(C–G)** Representative FACS plot (left) and quantification (right) of electroporated (ET, TOM^+*ve*^) single cells **(C)**, CD45^+*ve*^ **(D)**, CD31^+*ve*^ **(E)**, FAPs **(F)** and MuSCs **(G)** from *TA* of ∼5 months-old *C57BL/6J* mice injected with PBS (CNTR^–^ *^ve^*) or with tdTomato plasmid (ET) and electroporated. The analysis was performed 3 weeks after electroporation. *N* = 3. **(H–K; left)** Representative FACS plot showing in Q2 TOM^+*ve*^GFP^+*ve*^ cells in single cell **(H)**, CD45^+*ve*^CD31^+*ve*^ cell **(I)**, FAPs **(J)** and MuSCs **(K)** populations from *TA* of ∼4 months-old *C57BL/6J* mice electroporated with GFP/tdTomato plasmid mix and identified as effectively electroporated with tdTomato plasmid (ET) as shown in panels **(C–G)**. CNTR^–^ *^ve^* [shown only in **(H)**] represents non-electroporated cells of *TA* injected with PBS and electroporated. The analysis was performed 1 week after electroporation. Refer also to Supplementary Figures 3B–D for CNTR^–^ *^ve^* FACS plot of **(I–K)**. **(H–K; right)** Quantification of the GFP^+ve^ fraction of single cells **(H)**, CD45^+*ve*^CD31^+*ve*^ cells **(I)**, FAPs **(J)**, and MuSCs **(K)** effectively electroporated with tdTomato plasmid (ET) in *TA* of ∼4 months-old *C57BL/6J* mice injected with a GFP/tdTomato plasmids mix and electroporated. The analysis was performed 1 week after electroporation. *N* = 3. FAPs: CD45^–^ *^ve^*CD31^–^ *^ve^*sca1^+ve^ cells; MuSCs: CD45^–^ *^ve^*CD31^–^ *^ve^*sca1^–^ *^ve^*vcam^+*ve*^ cells.

A major aim of this work is to describe to which extent mononucleated cells present in skeletal muscles are susceptible to electro-enhanced plasmid transfer. With this goal we injected a GFP-expressing plasmid in the *TA* muscle of adult *C57BL/6J* mice, we electroporated the injected muscles, and we measured the GFP intensity through the Bruker *In Vivo* Xtreme I imaging system at different time points after the plasmid injection. In agreement with previous reports, this approach resulted in a strong GFP signal 1 week after plasmid delivery (Figure 1A and Supplementary Figure 2A), which indicated that the electroporation effectively led to the plasmids uptake in the muscles. Moreover, our *in vivo* imaging analysis showed a clear GFP signal in the electroporated *C57BL/6J* mice compared to the negative controls from 1.5 days up to 6 weeks after electroporation, indicating a long-term permanence of the plasmid in electroporated muscles (Supplementary Figures 2B,H). After having assessed that our *in vivo* electroporation effectively leads to the plasmid uptake in the hindlimb muscles, we aimed to get insights on the specific cells that are capable of receiving and expressing the electroporated plasmid. A histological analysis performed on muscle sections of electroporated *C57BL/6J* mice revealed effective plasmid uptake by muscle fibers and by nearby mononucleated cells (Figure 1B and Supplementary Figure 3A). A fraction of the targeted mononucleated cells resulted positive for the macrophage marker F/480. Nevertheless, numerous effectively electroporated mononucleated cells were F/480^–^*^ve^* (Figure 1B and Supplementary Figure 3A). To further investigate the nature of these cells, we injected a tdTomato-expressing plasmid in the *TA* of adult *C57BL/6J* and electroporated the same muscles. Three weeks after the electroporation, muscles were dissected and enzymatically digested to obtain a suspension of mononucleated cells. Mononucleated cells were stained with antibodies directed against surface markers for the identification of specific cell populations by FACS analysis (Supplementary Figure 1A). Our cytofluorimetric analysis revealed that up to 31.0% of the total mononucleated cells expressed the plasmid (Figure 1C). Within the single cells, up to 29.9% of CD45^+ve^ cells, 41.9% of CD31^+ve^ cells, 53.4% of FAPs and 25.5% of MuSCs expressed the electroporated plasmid (Figures 1D–G). Differences among subpopulations were not statistically significant (*p* > 0.05). Negligible fluorescence was observed in control muscles injected with PBS and electroporated (Figures 1C–G). Plasmid uptake by muscle mononucleated cells was clear as early as 1.5 days after electroporation (Supplementary Figures 2C–G). This analysis demonstrates that, in addition to syncytial muscle fibers, it is possible to effectively target various muscle mononucleated cells through electro-enhanced plasmid transfer.

To further characterize the properties of electro-enhanced plasmid transfer to muscle mononucleated cells, we evaluated the efficiency of plasmid uptake after simultaneous injection of two distinct plasmids. *In vitro* co-electroporation of myotubes with two different plasmids results in the preferential uptake of both plasmids by the same cells (Sandri et al., 2003). In order to assess whether this preferential uptake is occurring also in mononucleated cells after electroporation *in vivo*, we injected adult *C57BL/6J* muscles with a mix of GFP- and tdTomato- expressing plasmids. The FACS analysis performed 1 week after the electroporation revealed that 80.7 to 95.9% of TOM^+ve^ cells also expressed the GFP plasmid in all the analyzed cell populations (i.e., total single cells, CD45^+ve^CD31^+ve^ cells, FAPs and MuSCs), suggesting that those cells that are effectively electroporated have the tendency to uptake both plasmids (Figures 1H–K and Supplementary Figures 3B–D for negative controls).

### *In vivo* Electroporation of Injured Muscles

**FIGURE 2:**
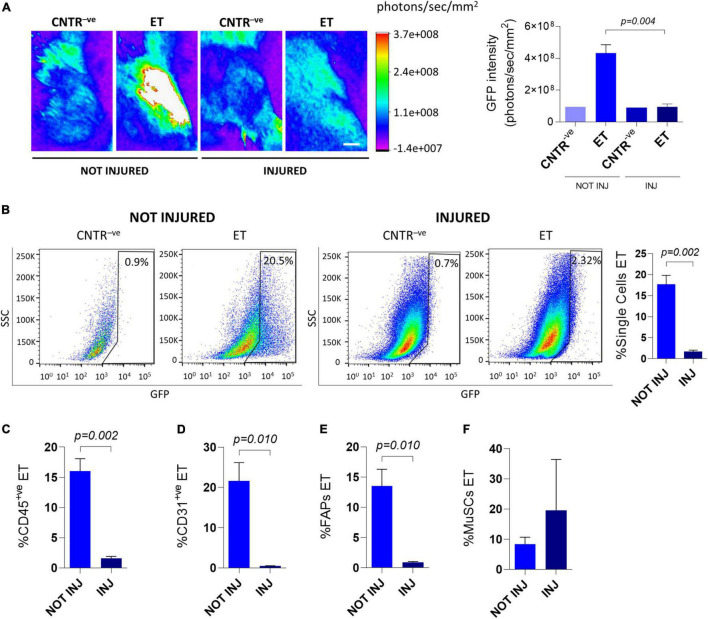
*In vivo* electroporation of injured muscles**. (A)** Representative Bruker *In Vivo* Xtreme I images (left) and GFP intensity measurement (photons/sec/mm^2^) (right) of *TA* muscles from ∼8 months-old *C57BL/6J* mice uninjured (NOT INJ) or injected with cardiotoxin (INJ). Mice were injected with PBS (CNTR^–^ *^ve^*) or GFP plasmid (ET) and electroporated 1.5 days after cardiotoxin injection. Scale bar: 2.5 mm. White color in the figure indicates photons/sec/mm^2^ > 3.7e+008, Muscles were analyzed 5 days after electroporation. *N* = 3 (ET), *N* = 1 (CNTR^–^ *^ve^*). **(B)** Representative FACS plot (left) and quantification (right) of electroporated (ET, GFP^+ve^) single cells from *TA* of ∼8 months-old *C57BL/6J* mice uninjured (NOT INJ) or injected with cardiotoxin (INJ). Mice were injected with PBS (CNTR^–^ *^ve^*) or GFP plasmid (ET) and electroporated 1.5 days after the cardiotoxin injection. The analysis was performed 6 days after electroporation. *N* = 3 (ET). **(C–F)** Quantification of electroporated (ET) CD45^+ve^ cells **(C)**, CD31^+ve^ cells **(D)**, FAPs **(E)** and MuSCs **(F)** from *TA* of the same mice as in panel **(B)**. All CNTR^–^ *^ve^* gates were set on control samples (i.e., sample injected with PBS and electroporated). The percentage of ET cells in each CNTR^–^ *^ve^* gate was < 1% for all the analyzed cell populations.

Pre-treatement with muscle-damaging agents reportedly reduces the efficiency of whole muscle electroporation (Gollins et al., 2003). To assess if tissue damage could also affect plasmid uptake by mononucleated cells, we electroporated *C57BL/6J* muscles with the GFP-expressing plasmid 1.5 days after intramuscular injection of cardiotoxin. As expected, 6 days after electroporation, the GFP intensity measured *in vivo* through the Bruker *In Vivo* Xtreme I imaging system was reduced in the cardiotoxin-treated mice compared to uninjured mice (Figure 2A). This protocol also resulted in a significant decrease in the electroporation efficiency of all analyzed mononucleated cell populations (i.e., total single cells, CD45^+ve^ cells, CD31^+ve^ cells, and FAPs) except MuSCs, for which the analysis gave variable results (Figures 2B–F). Intriguingly, a comparable low efficiency of electroporation (1.9 ± 1.0% for total single cells, 1.4 ± 0.6% for CD45^+ve^ cells, 2.3 ± 2.4% for CD31^+ve^ cells, 3.5 ± 3.6% for FAPs, and 1.9 ± 1.7% for MuSCs) was also observed when cardiotoxin was injected 1 week after electroporation and the FACS analysis was performed after 2 additional weeks. This observation suggests that the integrity of the muscle is crucial for the electroporation outcome.

### *In vivo* Electroporation of Dystrophic Muscles

**FIGURE 3:**
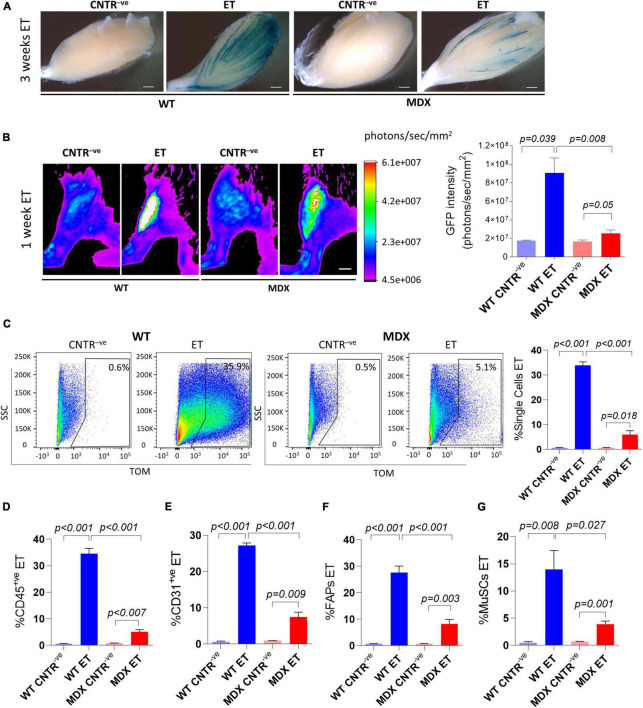
*In vivo* electroporation of dystrophic muscles. **(A)** Whole-mount X-gal staining of not electroporated (CNTR^–^ *^ve^*) and electroporated (ET) *TA* muscles from ∼16 months-old *C57BL/6J* (WT) and *mdx^4Cv^* (MDX) mice. β-gal plasmid was injected in muscles prior electroporation. Muscles were collected 3 weeks after electroporation. *N* = 2. Scale bar: 0.85 mm. **(B)** Representative Bruker *In Vivo* Xtreme I images (left) and GFP intensity measurement (photons/sec/mm^2^) (right) of *TA* muscles from 12–14 months-old *C57BL/6J* (WT) and *mdx^4Cv^* (MDX) mice injected with β-gal (CNTR^–^ *^ve^*) or GFP plasmid (ET) and electroporated. Scale bar: 2.5 mm. White color in the figure indicates photons/sec/mm^2^ > 6.1e+007. Muscles were analyzed 1 week after electroporation. *N* = 2 (WT CNTR^–^ *^ve^*), *N* = 4 (WT ET; MDX ET), *N* = 6 (MDX CNTR^–^ *^ve^*). **(C)** Representative FACS plot (left) and quantification (right) of electroporated (ET, TOM^+*ve*^ or GFP^+ve^) single cells from *TA* of ∼6–9 months-old *C57BL/6J* (WT) and *mdx^4Cv^* (MDX) mice injected with tdTomato or GFP plasmid and electroporated (ET). CNTR^–^ *^ve^* samples are hindlimb untreated muscles or muscles injected with PBS and electroporated. The analysis was performed 1 week after electroporation. *N* = 4. **(D–G)** Quantification of electroporated (ET) CD45^+ve^ cells (*N* = 3) **(D)**, CD31^+ve^ cells (*N* = 3) **(E)**, FAPs (*N* = 4) **(F)** and MuSCs (*N* = 4) **(G)** from muscles of the same mice as in panel **(C)**. All CNTR^–^ *^ve^* gates were set on control samples. The percentage of ET cells in each CNTR^–^ *^ve^* gate was < 1% for all the analyzed cell populations.

The *mdx^4Cv^* mouse model is one of the most widely used animal models to study Duchenne muscular dystrophy (DMD) (van Putten et al., 2020). Duchenne muscular dystrophy is characterized by chronic fiber necrosis and regeneration (Ciciliot and Schiaffino, 2010). Accumulation of fibrotic tissue characterizes *mdx^4Cv^* muscles as compared with age-matched controls, and collagen depositions is becoming conspicuous with age paralleling muscle deterioration (Pastoret and Sebille, 1995). Other than contributing to the dystrophic phenotype, muscle fibrosis potentially represents a physical barrier to the effective exogenous material uptake, including drugs and cells which are meant for the disease treatment (Gargioli et al., 2008). In order to assess if and how efficiently we were able to deliver an expression vector in the *mdx^4Cv^* muscles we injected a β-gal expressing plasmid (Figure 3A) and a GFP-expressing plasmid (Figure 3B) in adult *C57BL/6J* and *mdx^4Cv^* hindlimbs by applying the electroporation protocol. Our analysis of the GFP intensity performed 1 week after electroporation revealed clear GFP signals in both the *C57BL/6J* and the *mdx^4Cv^* muscles *in vivo* (Figure 3B). Intriguingly, the GFP intensity in the *mdx^4Cv^* mice was significantly reduced compared to the aged-matched *C57BL/6J* mice (Figure 3B). A similar trend was apparent also after whole–mount X-gal staining of muscles injected with β-gal-expressing plasmids and collected 3 weeks after electroporation (Figure 3A). The presence of individual fibers characterized by strong X-gal staining suggests that once plasmids are taken up by dystrophic fibers they might be maintained for several weeks (Figure 3A).

We next decided to investigate the extent of plasmid uptake in mononuclear cells by electroporating tdTomato-expressing plasmids in dystrophic muscles (Figures 3C–G). In keeping with our whole muscles’ analysis (Figures 3A,B) the percentage of cells which received the exogenous plasmid was significantly lower in *mdx^4Cv^* mice compared to the aged-matched *C57BL/6J* mice for all the analyzed cell populations. Up to 5.1% of the entire single cell population (Figure 3C), 6.0% of CD45^+ve^ cells (Figure 3D) 10.1% of CD31^+ve^ cells (Figure 3E), 12.0% of FAPs (Figure 3F) and 4.9% of MuSCs (Figure 3G) expressed the tdTomato vector after the electroporation in *mdx^4Cv^*. Altogether, these results indicate that the dystrophic muscle is intrinsically different in terms of efficiency of plasmid uptake upon electroporation.

**FIGURE 4:**
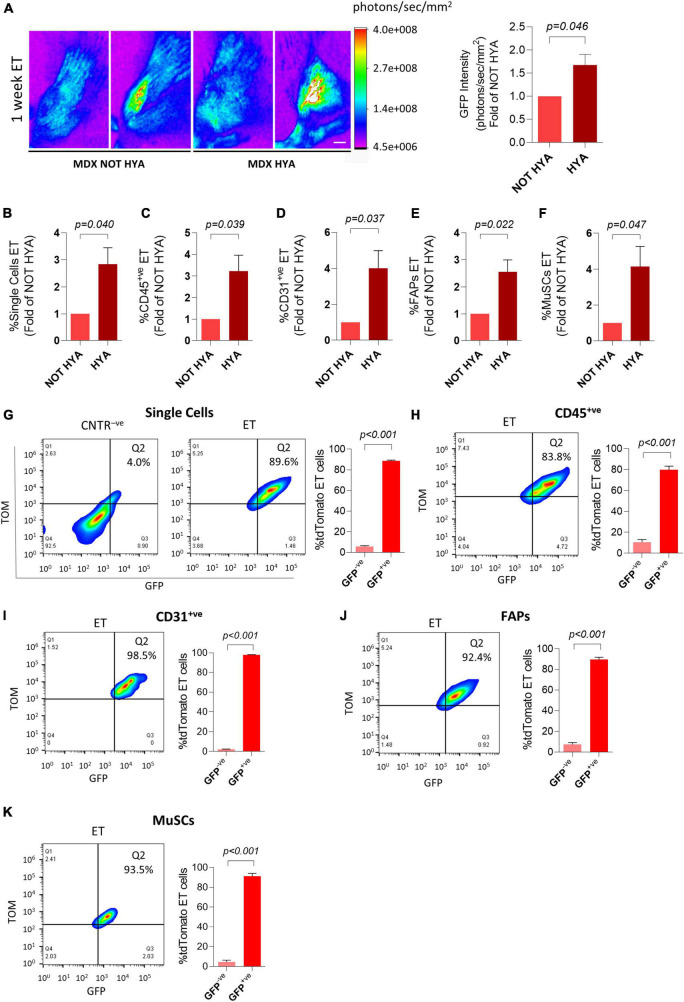
Hyaluronidase pre-conditioning increases the efficiency of electroporation of mononucleated cells in dystrophic muscles. **(A)** Representative Bruker *In Vivo* Xtreme I images (left) and GFP intensity measurement (photons/sec/mm^2^) (right) of *TA* muscles from ∼6 months-old *mdx^4Cv^* (MDX) mice injected with hyaluronidase (HYA) or not injected (NOT HYA). 2 h after hyaluronidase injection *TA* muscles were injected with PBS (CNTR^–^ *^ve^*) or GFP plasmid (ET) and electroporated. Scale bar: 2.5 mm. White color in the figure indicates photons/sec/mm^2^ > 4.0e+008. Muscles were analyzed 1 week after electroporation. *N* = 3. Refer also to Supplementary Figure 4A for GFP intensity measurement of the corresponding dissected muscles. **(B–F)** Quantification of electroporated (ET) single cells **(B)**, CD45^+ve^ cells **(C)**, CD31^+ve^ cells **(D)**, FAPs **(E)** and MuSCs **(F)** of *TA* muscles from the same mice as in panel **(A)**. All negative control gates were set on controls (i.e., samples treated or not treated with hyaluronidase, injected 2 h later with PBS and electroporated). The percentage of ET cells in each negative control gate was < 1% for all the analyzed cell populations. **(G–K; left)** Representative FACS plot showing in Q2 TOM^+ve^GFP^+ve^ cells in single cell **(G)**, CD45^+ve^ cell **(H)**, CD31^+ve^ cell **(I)**, FAPs **(J)**, and MuSCs **(K)** populations from *TA* of ∼6 months-old *mdx^4Cv^* (MDX) mice injected with hyaluronidase, and subsequently electroporated with GFP/tdTomato plasmid mix and identified as effectively electroporated with tdTomato plasmid (ET). CNTR^–^ *^ve^* [shown only in **(G)**] represents non-electroporated cells of *TA* treated with hyaluronidase, then injected with PBS and electroporated. The analysis was performed 1 week after electroporation. Refer also to Supplementary Figures 4B–E for CNTR^–^ *^ve^* FACS plots of **(H–K)**. **(G–K; right)** Quantification of the GFP^+ve^ fraction of single cells **(G)**, CD45^+ve^ cells **(H)**, CD31^+ve^ cells **(I)**, FAPs **(J)**, and MuSCs **(K)** effectively electroporated with tdTomato plasmid (ET) in *TA* of ∼6 months-old *mdx^4Cv^* (MDX) mice injected with hyaluronidase, then electroporated with GFP/tdTomato plasmid mix. The analysis was performed 1 week after electroporation. *N* = 3. FAPs: CD45^–^ *^ve^*CD31^–^ *^ve^*sca1^+ve^ cells; MuSCs: CD45^–^ *^ve^*CD31^–^ *^ve^*sca1^–^ *^ve^*vcam^+ve^ cells.

Hyaluronidase pre-treatment is reportedly increasing the efficiency of whole muscle electroporation both in *wild type* and *mdx* mice (McMahon et al., 2001; Gollins et al., 2003; Molnar et al., 2004). To assess if this approach could also increase plasmid uptake by mononucleated cells, we electroporated dystrophic muscles with plasmids expressing reporter genes after pre-conditioning with hyaluronidase. As expected, 1 week after electroporation, the GFP intensity measured *in vivo* and on dissected muscles through the Bruker *In Vivo* Xtreme I imaging system resulted higher in the hyaluronidase-treated mice compared to mice not injected with hyaluronidase (Figure 4A and Supplementary Figure 4A). Importantly, this protocol also resulted in a significant increase in the electroporation efficiency of mononucleated cells (Figures 4B–F). To further characterize the properties of electroporation in mononucleated cells of dystrophic tissues, we evaluated if the co-electroporation with two different plasmids results in the preferential uptake of both plasmids by the same cells as previously shown for *wild type* muscles. The FACS analysis performed 1 week after the electroporation with a mix of GFP- and tdTomato-expressing plasmids revealed that also in the *mdx^4Cv^* muscles, the great majority of TOM^+ve^ cells expressed GFP in all the analyzed cell populations (Figures 4G–K and Supplementary Figures 4B–E for negative controls). This aspect is not influenced by hyaluronidase, as similar results were obtained without hyaluronidase pre-conditioning (not shown). Altogether, these data highlight the possibility to effectively target the mononucleated cells in the dystrophic muscles.

### *In vivo* Electroporation of Old Muscles

**FIGURE 5:**
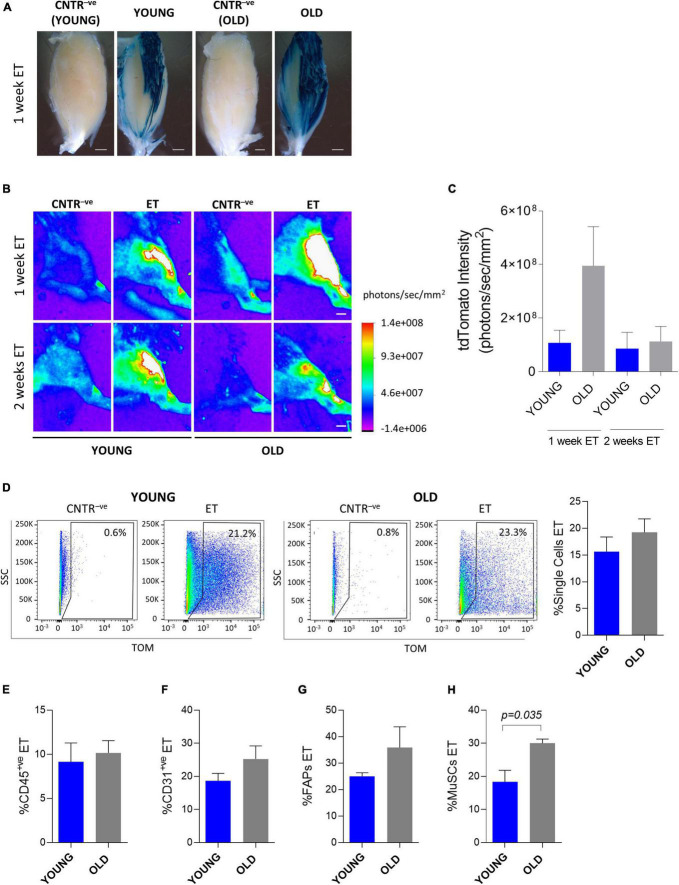
*In vivo* electroporation of old muscles. **(A)** Whole-mount X-gal staining of *TA* from ∼2 months-old *C57BL/6J* (YOUNG), and ∼24 months-old *C57BL/6J* (OLD) mice. PBS (CNTR^–^ *^ve^*) or β-gal plasmid (YOUNG, OLD) were injected in muscles prior electroporation. Muscles were collected 1 week after electroporation. Scale bar: 0.85 mm. **(B)** Representative Bruker *In Vivo* Xtreme I images of tdTomato intensity (photons/sec/mm^2^) of *TA* from muscles of ∼2 months-old (YOUNG) and ∼24 months-old (OLD) *C57BL/6J* mice injected with PBS (CNTR^–^ *^ve^*) or with tdTomato plasmid (ET) and electroporated. Scale bar: 2.5 mm. White color in the figure indicates photons/sec/mm^2^ > 1.4e+008. The analysis was performed 1 and 2 weeks after electroporation. **(C)** tdTomato intensity measurement (photons/sec/mm^2^) of *TA* of ∼2 months-old (YOUNG) and ∼24 months-old (OLD) *C57BL/6J* mice injected with tdTomato plasmid and electroporated. The analysis was performed 1 and 2 weeks after electroporation. *N* = 6 (YOUNG ET 1 week, OLD ET 1 week), *N* = 3 (YOUNG ET 2 weeks, OLD ET 2 weeks). The intensity of tdTomato of the corresponding CNTR^–^ *^ve^* samples (i.e., mice injected with PBS and electroporated) was subtracted from each sample. **(D)** Representative FACS plot (left) and quantification (right) of electroporated (ET) single cells from *TA* of ∼2 months-old (YOUNG) and ∼24 months-old (OLD) *C57BL/6J* mice injected with tdTomato plasmid and electroporated. The analysis was performed 2 weeks after electroporation. *N* = 3. **(E–H)** Quantification of electroporated (ET) CD45^+ve^ cells **(E)**, CD31^+ve^ cells **(F)**, FAPs **(G)** and MuSCs **(H)** from *TA* of the same mice as in panel **(D)**. All CNTR^–^ *^ve^* gates were set on control samples (i.e., sample injected with PBS and electroporated). The percentage of ET cells in each CNTR^–^ *^ve^* gate was < 1% for all the analyzed cell populations. FAPs: CD45^–^ *^ve^*CD31^–^ *^ve^*sca1^+ve^ cells; MuSCs: CD45^–^ *^ve^*CD31^–^ *^ve^*sca1^–^ *^ve^*vcam^+ve^ cells.

Both dystrophic and aging muscles are characterized by excessive accumulation of fibrotic tissue, but only dystrophic tissues are actively regenerating in the absence of external damaging events. To get insights into the relative contribution of fibrosis and regeneration-associated cellular turnover to the reduced electroporation efficiency observed in dystrophic muscles, we directed our attention to old mice. We evaluated the efficiency of *in vivo* plasmid electroporation by analyzing both whole muscles and muscle mononucleated cells in old (∼2 years-old) and young (∼2 months-old) *C57BL/6J* mice (Figure 5). Expression from a β-gal-expressing plasmid was readily observed 1 week after electroporation at both ages (Figure 5A). Interestingly, the β-gal expression appeared more intense in the old muscles compared to the young mice (Figure 5A). This observation was confirmed by an independent experiment in which a tdTomato-expressing vector was electroporated. Although the difference was not statistically significant, 1 week after electroporation the tdTomato intensity measured through the Bruker *In Vivo* Xtreme I imaging system resulted slightly higher in the old mice compared to the young mice (Figures 5B,C). Two weeks after the electroporation the plasmid was still expressed in both old and young muscles, but at comparable levels (Figures 5B,C). The FACS analysis showed that the electroporated vector was effectively delivered in the muscle mononucleated cells in both young and old muscles (Figures 5D–H). Two weeks after the *in vivo* electroporation 12.4 to 23.3% of the whole mononucleated cell population (Figure 5D), 6.7 to 13.4% of CD45^+ve^ population (Figure 5E), 14.8 to 32.5% of CD31^+ve^ population (Figure 5F), 23.6 to 48.8% of FAPs (Figure 5G) and 11.7 to 31.6% of MuSCs (Figure 5H) were effectively expressing the vector in both the young and old muscles. Altogether, these data indicate that despite the presence of fibrosis, the efficiency of electro-enhanced plasmid transfer in old muscle is not reduced compared to the situation in young adults.

### Study of Collagen 1 Expression in Muscle Single Cells Through *in vivo* Electroporation

**FIGURE 6:**
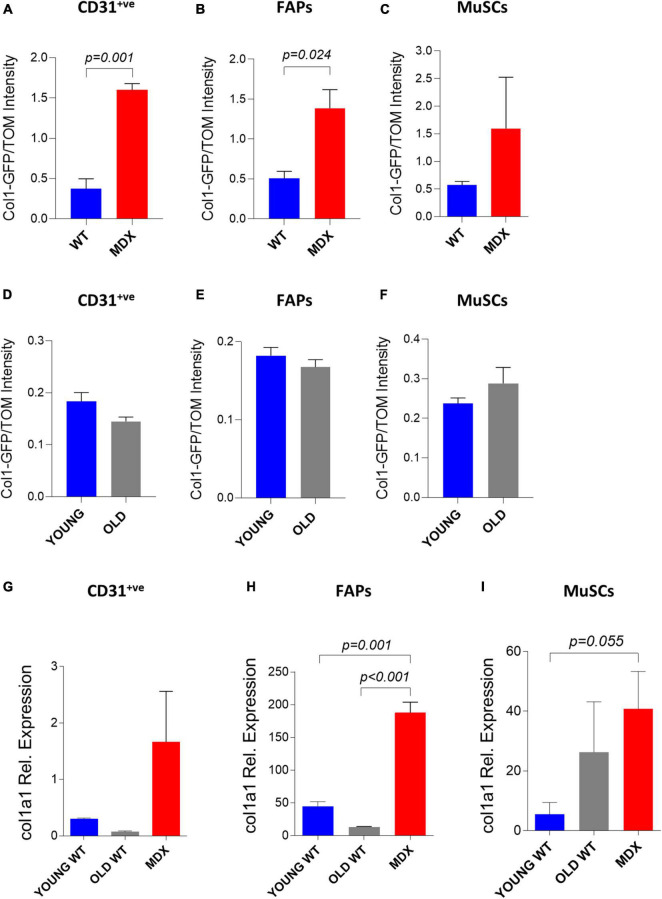
Study of collagen 1 expression in muscle single cells through *in vivo* electroporation. **(A–C)** Col1-GFP/TOM Intensity (median) in CD31^+*ve*^ **(A)**, FAPs **(B)** and MuSCs **(C)** from *TA* of ∼12 months-old *C57BL/6J* (WT) and *mdx^4Cv^* (MDX) mice injected with Col1-GFP:tdTomato (Col1-GFP/TOM) mix (10:1) and electroporated. The analysis was performed 2 weeks after electroporation. *N* = 3 for all samples except MuSCs MDX (*N* = 2). **(D–F)** Col1-GFP/TOM Intensity (median) in CD31^+*ve*^ **(D)**, FAPs **(E)** and MuSCs **(F)** from *TA* of ∼2 months-old (YOUNG) and ∼24–26 months-old (OLD) *C57BL/6J* mice injected with Col1-GFP/TOM mix (10:1) and electroporated. The analysis was performed 2 weeks after electroporation. *N* = 3. **(G–I)** qPCR analysis of *collagen1a1* in CD31^+*ve*^ **(G)**, FAPs **(H)** and MuSCs **(I)** from ∼2 months-old *C57BL/6J* (YOUNG WT), ∼24 months-old (OLD WT) and ∼12 months-old *mdx^4Cv^* (MDX) hindlimb muscles. FAPs: CD45^–^ *^ve^*CD31^–^ *^ve^*sca1^+ve^ cells; MuSCs: CD45^–^ *^ve^*CD31^–^ *^ve^*sca1^–^ *^ve^*vcam^+ve^ cells.

Our data indicate that the electroporation can deliver with different yet satisfactory efficiency an exogenous DNA vector in both healthy and compromised (i.e., dystrophic and old) muscles at the mononucleated cell level. This possibility opens to the *in vivo* investigation of the behavior of muscle mononucleated cells with unprecedented simplicity. As a proof of principle, we decided to take advantage of the electroporation protocol to investigate the involvement of various mononucleated cells to the accumulation of fibrotic tissue in dystrophic and aging muscle. Collagens are one of the major components of muscle extracellular matrix and, within all the different members of collagen superfamily, collagen 1 is the most abundant in the skeletal muscles (McKee et al., 2019). With the goal to study collagen 1 expression in muscle mononucleated cells we used the *in vivo* electroporation of a plasmid expressing the reporter gene GFP under the control of a portion of the collagen 1 promoter (Col1-GFP) (Kalajzic et al., 2002). Our observation indicating that two plasmids can be efficiently co-electroporated in the same cells in both dystrophic and *wild type* muscles (see above) allowed the use of a second plasmid expressing tdTomato under the control of the constitutive CMV promoter to normalize for the differences in the efficiencies of DNA transfer between dystrophic and *wild type* muscles. We therefore electroporated a mix of Col1-GFP and tdTomato plasmids in *TA* muscles of age-matched *C57BL/6J* and *mdx^4Cv^* mice. Two weeks after electroporation the muscles were dissected and digested to mononucleated cells. The GFP and tdTomato fluorescence intensities were measured by FACS. The level of GFP expression from the collagen 1 promoter was higher in the CD31^+ve^ cells (Figure 6A) and FAPs (Figure 6B) of the *mdx^4Cv^* muscles compared to the *C57BL/6J* after normalization with the corresponding tdTomato intensity. A similar trend, although not statistically significant, was also observed for MuSCs (Figure 6C). To corroborate these results, we investigated in parallel the mRNA levels of *collagen1a1* by quantitative PCR. Overall, the quantification of *collagen1a1* transcripts paralleled the results obtained with the plasmid expressing the reporter from the collagen promoter, although the difference was statistically significant only for FAPs (Figures 6G–I). This observation is in keeping with data reported in previous studies (Biressi et al., 2014; Pessina et al., 2015), and therefore confirmed the suitability of our electroporation-based approach to investigate the features of mononuclear cells in dystrophic tissues.

The same experiment was also performed to compare young and old *C57BL/6J* muscle mononucleated cells. We did not observe a significant increase in GFP expression from the *collagen 1* promoter in the old CD31^+ve^ cells (Figure 6D), FAPs (Figure 6E), and MuSCs (Figure 6F) compared to their young counterparts. In line with these results, our mRNA expression analysis on FACS-isolated CD31^+ve^ cells (Figure 6G) and FAPs (Figure 6H) from old and young muscles showed similar expression of *collagen1a1*. A not statistically significant trend toward rising levels of *collagen 1a 1* expression was observed in MuSCs from old muscles (Figure 6I). Altogether, these results demonstrate the feasibility of targeting muscle mononucleated cells through electro-enhanced plasmid transfer to investigate the cellular and molecular events characterizing diseased muscle tissues.

## Discussion

The data presented here highlight previously unappreciated features of the *in vivo* electroporation technique, focusing on the mononucleated cells. By taking advantage of a widely used set of surface markers, we unambiguously identified MuSCs, FAPs, endothelial cells, and hematopoietic lineage cells in electroporated murine muscles and comparatively quantified their efficiencies of electroporation. Transgene expression from transferred plasmid DNA was effectively induced in all these cell populations. These results are in keeping with previous observations reporting effective plasmid uptake by Vimentin^+ve^, Desmin^+ve^, or MyoD^+ve^ “bona fide” MuSCs in electroporated muscles (Grønevik et al., 2003; Peng et al., 2005; Ratanamart et al., 2010). To our knowledge, this is the first study conclusively demonstrating that electroporation can effectively target both endothelial cells and FAPs. A previous report indicates the existence of rare electroporated Thy1^+ve^ muscle interstitial cells, but due to the expression of this marker in various cell types, it was not possible to unambiguously establish their nature (Grønevik et al., 2003). Past studies investigated the plasmid transfer in hematopoietic lineage cells with conflicting results. Differences in the electroporation protocols, the spe cific markers used to identify the cells, and the sensitivity of the techniques employed to evaluate plasmid uptake and transgene expression may contribute to these discrepancies. Some studies reported undetectable levels of transgene expression by lymphocytes and macrophages/monocytes upon electro-enhanced plasmid transfer (Dupuis et al., 2000; Grønevik et al., 2003; Ratanamart et al., 2010). Nevertheless, one of these studies demonstrated that mononucleated cells expressing the myeloid marker CD11b are capable of efficiently uptaking electroporated plasmid DNA, possibly resulting from phagocytosis events (Dupuis et al., 2000). Intriguingly, intramuscular DNA injection reportedly induces transgenic protein expression in cells isolated from the proximal draining lymph nodes and mainly consisting in macrophages (Chattergoon et al., 1998). Our analysis indicating that CD45^+ve^ cells from electroporated muscles, particularly the F4/80^+ve^ macrophage subset, are capable of transgene expression is in line with this latter study. It is unknown if this is the result of the direct uptake of plasmids or depends on the high phagocytic activity of these cells.

We then extended our analysis of the electroporated mononucleated cells to dystrophic muscle. We were able to easily identify electroporated MuSCs, but we also noticed a clear reduction in the efficiency of electroporation. This reduction might explain why, despite effective electroporation in the mature fibers, only rare plasmid uptake was reported in dystrophic MuSCs in a previous study (Wong et al., 2005). In apparent contrast with this latter study, our observations highlight previously unappreciated parallelism between plasmid uptake by MuSCs and muscle fibers, being both significantly reduced after electroporation in dystrophic muscle compared to age-matched controls. A reduction in muscle fibers’ electroporation efficiency in *mdx* mice finds confirmation in a previous report (Molnar et al., 2004), which is inconsistent with others reporting comparable levels between *mdx* and *wild type* mice (Gollins et al., 2003). Importantly, we report compelling evidence indicating that hematopoietic cells, endothelial cells and FAPs from dystrophic mice are also less susceptible to electro-enhanced plasmid transfer, similar to their MuSCs and fiber counterparts.

Dystrophic muscle is characterized by chronic cycles of degeneration and regeneration and pronounced deposition of fibrotic tissue, all features that might potentially impact electroporation efficiency. Fibrosis might represent a physical barrier for intramuscular diffusion but is likely not the major cause of ineffective plasmid uptake that we observed in *mdx* mice. Indeed, our data indicate that electroporation efficiency is not decreasing in 2 years-old muscles, which are also characterized by fibrosis. The idea of little influence of fibrosis finds further confirmation in the observation that electroporation efficacy is not affected by the increased amount of collagen present in *mdx^4Cv^* muscles at 21 weeks of age compared to the situation in 7 weeks-old mice (Gollins et al., 2003). MuSCs, FAPs, hematopoietic and endothelial cells are all undergoing a dramatic expansion in dystrophic tissues as part of the ongoing regenerative response (Wosczyna and Rando, 2018). The possibility that proliferating cells might be less effective in uptaking plasmids than their quiescent counterpart might explain why efficiencies are higher in all cell populations when non-dystrophic tissues are electroporated. The mechanism underlining this aspect may reside in the observation that electro-enhanced plasmid transfer depends on the radius of curvature of the target cell (Somiari et al., 2000). Indeed, cells undergo a dramatic change in size and morphology as they transit from quiescence to proliferation during regeneration (van Velthoven and Rando, 2019). Moreover, proliferating cells are also less resistant than quiescent cells to a variety of stressors, and this might also contribute to the overall efficiency, as electroporation is inevitably associated with a mild degree of damage (Roche et al., 2011). Newly repaired fibers derive from the fusion of activated MuSCs, and a causal link between the efficacy of plasmid uptake by MuSCs and the efficacy of electroporation in regenerating fibers has been proposed in *wild type* muscles and is indirectly supported by our data in the dystrophic setting (Peng et al., 2005). Hyaluronidase remodels the extracellular matrix and protects muscle from damage (McMahon et al., 2001). By taking these aspects into account, the observation reported here that the pretreatment with hyaluronidase increases electroporation efficiency both in whole muscle and in mononucleated cells fits with the hypothesis that activated cells are less prone to uptake DNA or present a higher tendency to lose plasmids upon electroporation. This idea is also indirectly confirmed by actively growing 2-3 weeks-old *mdx* mice, which are less efficiently electroporated than their 6 weeks-old adult counterparts (Molnar et al., 2004). Furthermore, previous reports demonstrate that the administration of damaging agents a few days before electroporation, which promotes fibers degeneration and subsequent proliferative expansion of mononucleated cells, also reduces whole muscle electroporation efficiencies (Gollins et al., 2003; Wong et al., 2005). Here we have not only extended this observation to various mononucleated cells, corroborating the parallelism between fibers and mononucleated cells electroporation, but we also have noticed poor incorporation of plasmids in mononucleated cells when the damaging agent was administered after the electroporation procedure (see above). This latter observation suggests that proliferating cells might dilute out electroporated non-integrating plasmids to result in fewer positive cells as the population expands after transfection. Therefore, although concomitant factors such as fibrosis may also play a role, the proliferative state of the mononucleated cells appears crucial to define the outcome of the electro-enhanced plasmid transfer.

Electroporation is a cost-effective procedure that might represent a valuable alternative to viral vectors, which may be troublesome to handle on the laboratory scale. Our observation that various subpopulations of mononucleated cells involved in skeletal muscle repair are amenable for electro-enhanced plasmid transfer is opening new investigative scenarios extending beyond replacement gene transfer and systemic production of therapeutic proteins that are classically accomplished through muscle fiber electroporation. Overexpression or silencing of genes potentially involved in modulating the lineage progression of MuSCs, hematopoietic cells, endothelial cells, and FAPs may represent a powerful tool to investigate the fundamental mechanisms controlling regeneration and get insight into cellular and molecular events, which are getting defective during aging and muscle diseases. The incomplete efficiency of electroporation, particularly for the dystrophic and injured muscles, might represent a limitation to this approach that may be at least in part overcome by introducing reporter genes in the electroporated plasmids to identify the fraction of effectively targeted cells in the tissue. Another limitation is that electroporation is not readily applicable to non-limb muscles, such as the diaphragm, which is diffusely used to model disease progression in DMD animal models (Stedman et al., 1991).

Electro-enhanced plasmid transfer to mononucleated cells is also rendering possible to investigate *in vivo* and in a cell-specific way the function of regulatory DNA sequences involved in the transcriptional control of specific genes without the need to turn to the generation of engineered mouse models. This possibility is highlighted by the study we are reporting here, in which a portion of 2.3 kb of the *collagen1a1* promoter was shown to recapitulate endogenous *collagen1a1* transcription in dystrophic mononuclear cells. *In vivo* electro-enhanced plasmid delivery is also attractive in gene editing experiments. The episomal nature of electroporated plasmids favors a transient “hit-and-run” approach that minimizes off-targets and undesired immune responses (Mashel et al., 2020).

We also report the possibility to effectively co-transfer multiple plasmids in the same cells by electroporation. As we show here, this feature can be exploited for normalization purposes but is potentially useful in other experimental settings. For instance, multiple gene transfer may permit the simultaneous modulation of concurrent biochemical pathways and may be exploited in basic research and therapy (Daddi et al., 2002). Co-delivery of different expression plasmids can be practical when a single plasmid expressing all the genes of interest is not available or, if generated, would result in a large construct, which can negatively affect cellular uptake. Co-electroporation is also potentially useful in studies involving CRISPR/Cas9-mediated genetic modifications, where the definition of the optimal ratio between guide RNA and Cas9 expression is required for efficient gene editing. The use of two independent plasmids, respectively expressing the guide RNA and Cas9 might facilitate the process of optimization (Gam et al., 2019).

An increasing amount of publications highlights the primary involvement of MuSCs, and potentially also of the other muscle mononucleated cells, in the etiology of diseases previously believed to depend exclusively on muscle fibers defects (Ganassi et al., 2021). The observation that mononucleated muscle cell populations may be easily targeted *in vivo* with a versatile protocol such as electroporation offers a new weapon in the hands of researchers interested in investigating their properties and functions.

## Data Availability Statement

The raw data supporting the conclusions of this manuscript will be made available by the authors, without undue reservation, to any qualified researcher.

## Ethics Statement

The animal experiments have been approved by the institutional animal use and welfare committee and the Italian Ministry of Health.

## Author Contributions

FF, SA, ML, and SB conducted and interpreted the experiments. FF and SB contributed to the design of the experiments and wrote the manuscript. All authors contributed to the article and approved the submitted version.

## Conflict of Interest

The authors declare that the research was conducted in the absence of any commercial or financial relationships that could be construed as a potential conflict of interest.

## Publisher’s Note

All claims expressed in this article are solely those of the authors and do not necessarily represent those of their affiliated organizations, or those of the publisher, the editors and the reviewers. Any product that may be evaluated in this article, or claim that may be made by its manufacturer, is not guaranteed or endorsed by the publisher.

## Footnotes

1 http://n2t.net/addgene:110210; RRID:Addgene_110210

## Supplementary Material

Supplementary Figure 1 Gating strategy used to isolate single cell populations. **(A)** Representative gating and sorting strategy used to FACS isolate CD45^+ve^, CD31^+ve^, FAPs (CD45^–^*^ve^*CD31^–^*^ve^*sca1^+ve^) and MuSCs (CD45^–^*^ve^*CD31^–^*^ve^*sca1^–^*^ve^*vcam^+ve^) population from *C57BL/6J* and *mdx^4Cv^* hindlimb muscles.

Supplementary Figure 2 *In vivo* electroporation of whole muscles and single cells. **(A)** Reflectance images of untreated *gastrocnemius* (CNTR^–^*^ve^*) and electroporated *TA* (ET) shown in Figure 1A. Scale bar: 2.5 mm. **(B)** Representative Bruker *In Vivo* Xtreme I images (left) and GFP intensity measurement (photons/sec/mm^2^) (right) of *TA* muscles from ∼14 months-old *C57BL/6J* mice injected with GFP plasmid and electroporated (ET) or not treated (CNTR^–^*^ve^*). Scale bar: 2.5 mm. White color in the figure indicates photons/sec/mm^2^ > 3.7e+008. The analysis was performed 1.5 day after electroporation. **(C–G)** Representative FACS plot (left) and quantification (right) of electroporated (ET, GFP^+ve^) single cells **(C)**, CD45^+ve^ cells **(D)**, CD31^+ve^ cells **(E)**, FAPs **(F)** and MuSCs **(G)** from *TA* of ∼14 months-old *C57BL/6J* mice injected with PBS (CNTR^–^*^ve^*) or with GFP-expressing plasmid (ET) and electroporated. The analysis was performed 1.5 day after electroporation. *N* = 3 (ET), *N* = 1 (CNTR^–^*^ve^*). FAPs: CD45^–^*^ve^*CD31^–^*^ve^*sca1^+ve^ cells; MuSCs: CD45^–^*^ve^*CD31^–^*^ve^*sca1^–^*^ve^*vcam^+ve^ cells. **(H)** Representative Bruker *In Vivo* Xtreme I images (left) and GFP intensity measurement (photons/sec/mm^2^) (right) of *TA* muscles from ∼13 to 14 months-old *C57BL/6J* mice injected with β-gal (CNTR^–^*^ve^*) or GFP plasmid (ET) and electroporated. Scale bar: 2.5 mm. White color in the figure indicates photons/sec/mm^2^ > 1.5e+008. The analysis was repeated 1, 2, 4, and 6 weeks (w) after electroporation. *N* = 4 (CNTR^–^*^ve^* 1w, ET 4w), *N* = 5 (ET 1w), *N* = 1 (CNTR^–^*^ve^* 2w, ET 2w, ET 6w).

Supplementary Figure 3 Histological analysis of electroporated muscles. **(A)** Immunofluorescence images of *TA* from additional ∼8 months-old *C57BL/6J* mice injected with GFP plasmid, electroporated and processed as described in Figure 1B. Muscles were stained with anti-GFP (green), anti-F4/80 (orange) antibodies, and Hoechst (blue). BF: Bright Field. Scale bar: 10 μm. Note the presence of both electroporated (GFP^+ve^) F4/80^+ve^ cells (red asterisks) and F4/80^–^*^ve^* mononucleated muscle cells (red arrows). White asterisks and arrows are, respectively, marking examples of non-electroporated (GFP^–^*^ve^*) F4/80^+ve^ and F4/80^–^*^ve^* mononucleated muscle cells. **(B–D)** CNTR^–^*^ve^* FACS plot referred to Figures 1I–K showing CD45^+ve^CD31^+ve^ cells **(I)**, FAPs **(J)**, and MuSCs **(K)** of *TA* of ∼4 months-old *C57BL/6J* mice injected with PBS and electroporated.

Supplementary Figure 4 Hyluronidase treatement in dystrophic muscles. **(A)** Representative Bruker *In Vivo* Xtreme I images (left) and GFP intensity measurement (photons/sec/mm^2^) (right) of dissected *TA* from ∼6 months-old *mdx^4Cv^* (MDX) mice injected with hyaluronidase (HYA) or not injected (NOT HYA). 2 h after hyaluronidase injection *TA* muscles were injected with PBS (CNTR^–^*^ve^*) or GFP plasmid (ET) and electroporated. Scale bar: 2.5 mm. White color in the figure (left, top panel) indicates photons/sec/mm^2^ > 1.6e+010. Corresponding reflectance images are shown in the bottom panel. Muscles were analyzed 1 week after electroporation. *N* = 3. **(B–E)** CNTR^–^*^ve^* FACS plots referred to Figures 4H–K showing CD45^+ve^ cells **(B)**, CD31^+ve^ cells **(C)**, FAPs **(D)**, and MuSCs **(E)** of *TA* of ∼6 months-old *mdx^4Cv^* mice *C57BL/6J* mice injected with hyaluronidase, then injected with PBS and electroporated.
