# Supplementary material for: Targeting Muscle-Resident Single Cells Through in vivo Electro-Enhanced Plasmid Transfer in Healthy and Compromised Skeletal Muscle
Source: Front Physiol. 2022 Apr 1;13:834705. doi: 10.3389/fphys.2022.834705 (PMC9010744; doi:10.3389/fphys.2022.834705)
Supplement: Supplementary file 1 [file Table_1.DOCX]

**Supplementary Table 1.** Primary antibodies used to FACS isolate muscle cells.

| **Antibody** | **Company** | **Dilution** |
| --- | --- | --- |
| APC/Fire 750 anti-mouse CD45 | BioLegend (103154) | 1:100 |
| APC/Fire 750 anti-mouse CD31 | BioLegend (102434) | 1:100 |
| PerCP/Cy5.5 anti-mouse CD31 | BioLegend (102420) | 1:100 |
| Brillant Violet 421 anti-mouse Ly-6A/E (Sca1) | BioLegend (108127) | 1:100 |
| Biotin anti-mouse CD016 (Vcam) | BioLegend (105704) | 1:100 |
