# Supplementary figures and images for: Targeting Muscle-Resident Single Cells Through in vivo Electro-Enhanced Plasmid Transfer in Healthy and Compromised Skeletal Muscle
Source: Front Physiol. 2022 Apr 1;13:834705. doi: 10.3389/fphys.2022.834705 (PMC9010744; doi:10.3389/fphys.2022.834705)

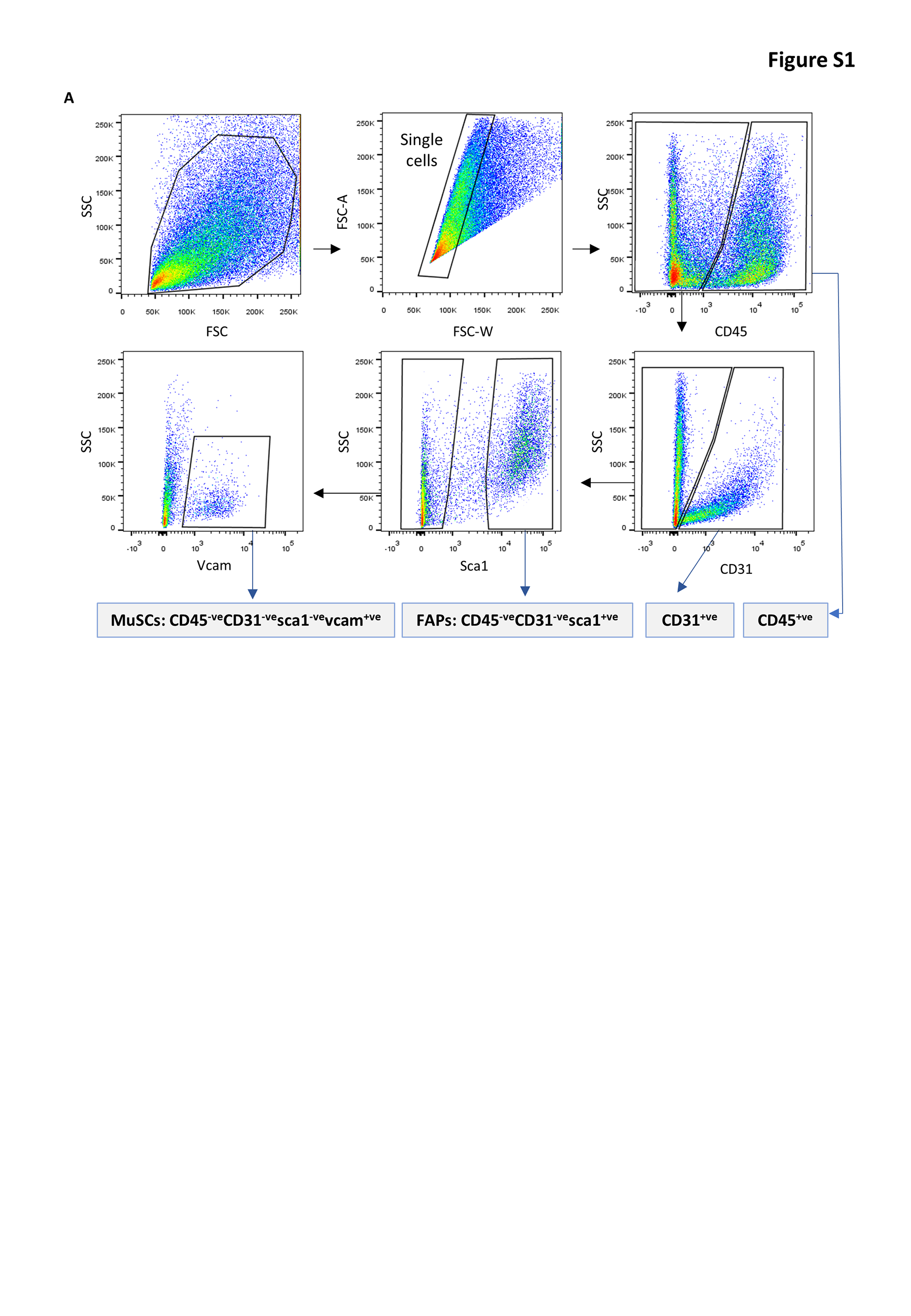

Supplement: Supplementary Figure 1 — Gating strategy used to isolate single cell populations. (A) Representative gating and sorting strategy used to FACS isolate CD45+ve, CD31+ve, FAPs (CD45–veCD31–vesca1+ve) and MuSCs (CD45–veCD31–vesca1–vevcam+ve) population from C57BL/6J and mdx4Cv hindlimb muscles. [file Image_1.TIF]

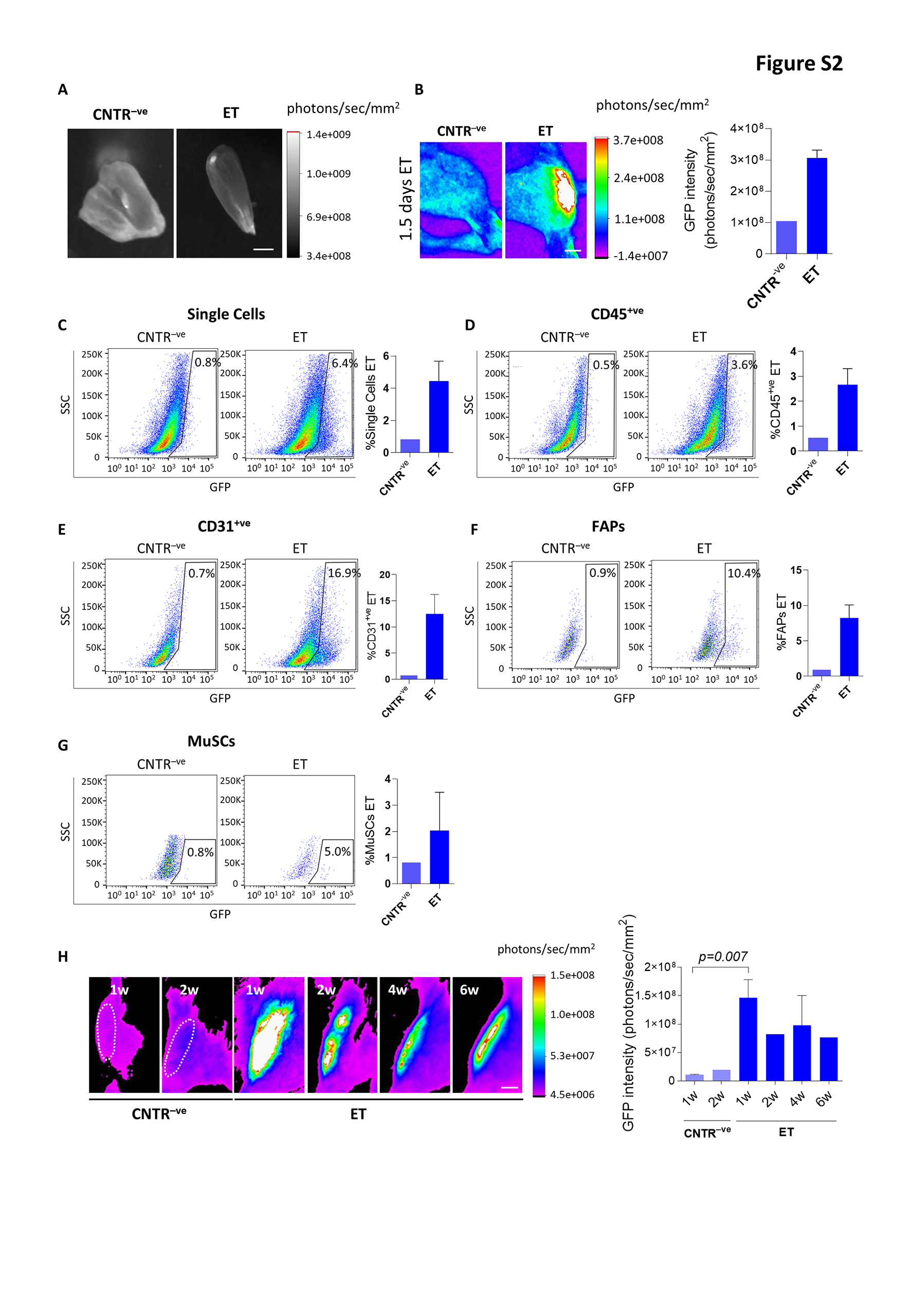

Supplement: Supplementary Figure 2 — In vivo electroporation of whole muscles and single cells. (A) Reflectance images of untreated gastrocnemius (CNTR–ve) and electroporated TA (ET) shown in Figure 1A. Scale bar: 2.5 mm. (B) Representative Bruker In Vivo Xtreme I images (left) and GFP intensity measurement (photons/sec/mm2) (right) of TA muscles from ∼14 months-old C57BL/6J mice injected with GFP plasmid and electroporated (ET) or not treated (CNTR–ve). Scale bar: 2.5 mm. White color in the figure indicates photons/sec/mm2 > 3.7e+008. The analysis was performed 1.5 day after electroporation. (C–G) Representative FACS plot (left) and quantification (right) of electroporated (ET, GFP+ve) single cells (C), CD45+ve cells (D), CD31+ve cells (E), FAPs (F) and MuSCs (G) from TA of ∼14 months-old C57BL/6J mice injected with PBS (CNTR–ve) or with GFP-expressing plasmid (ET) and electroporated. The analysis was performed 1.5 day after electroporation. N = 3 (ET), N = 1 (CNTR–ve). FAPs: CD45–veCD31–vesca1+ve cells; MuSCs: CD45–veCD31–vesca1–vevcam+ve cells. (H) Representative Bruker In Vivo Xtreme I images (left) and GFP intensity measurement (photons/sec/mm2) (right) of TA muscles from ∼13 to 14 months-old C57BL/6J mice injected with β-gal (CNTR–ve) or GFP plasmid (ET) and electroporated. Scale bar: 2.5 mm. White color in the figure indicates photons/sec/mm2 > 1.5e+008. The analysis was repeated 1, 2, 4, and 6 weeks (w) after electroporation. N = 4 (CNTR–ve 1w, ET 4w), N = 5 (ET 1w), N = 1 (CNTR–ve 2w, ET 2w, ET 6w). [file Image_2.TIF]

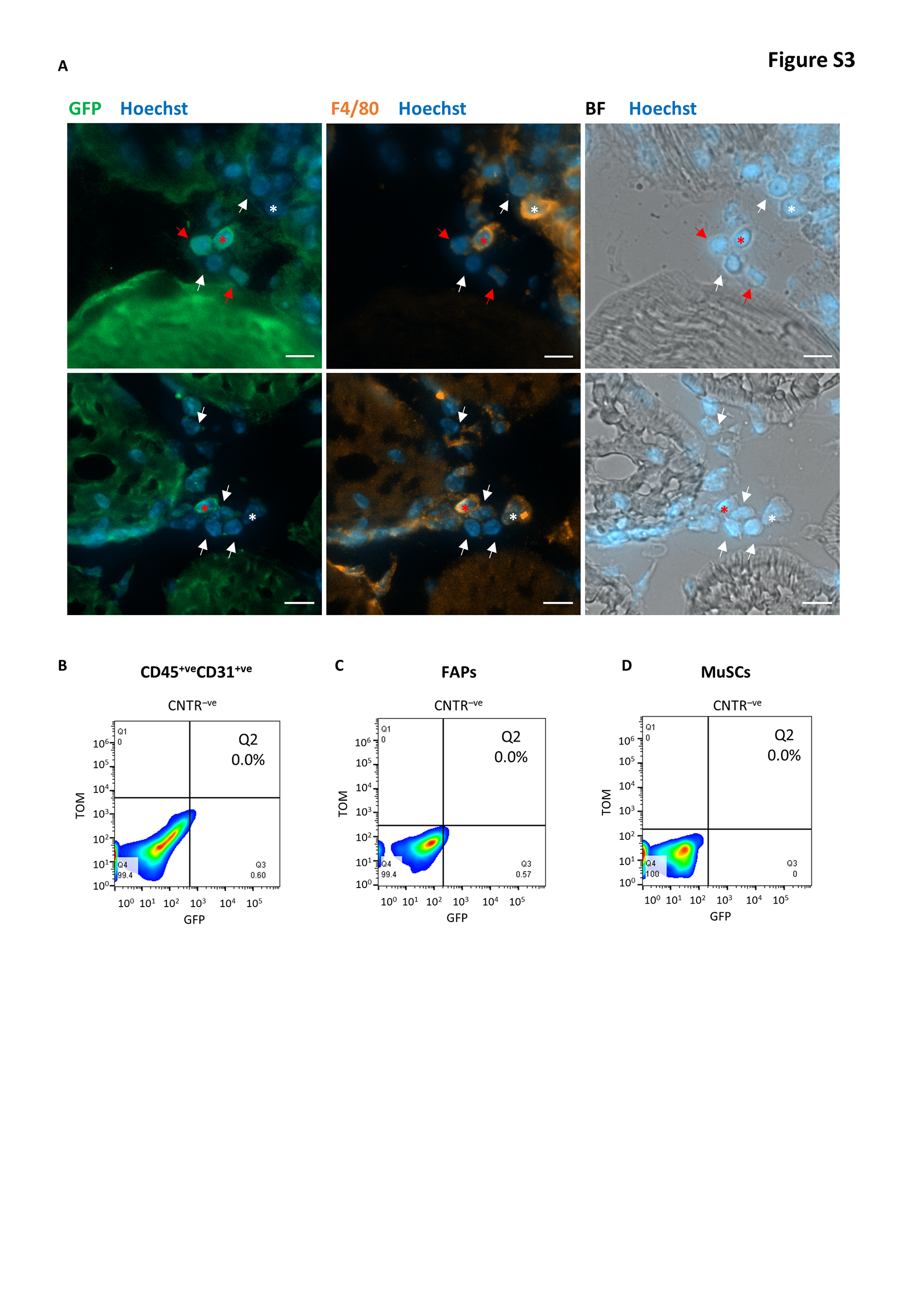

Supplement: Supplementary Figure 3 — Histological analysis of electroporated muscles. (A) Immunofluorescence images of TA from additional ∼8 months-old C57BL/6J mice injected with GFP plasmid, electroporated and processed as described in Figure 1B. Muscles were stained with anti-GFP (green), anti-F4/80 (orange) antibodies, and Hoechst (blue). BF: Bright Field. Scale bar: 10 μm. Note the presence of both electroporated (GFP+ve) F4/80+ve cells (red asterisks) and F4/80–ve mononucleated muscle cells (red arrows). White asterisks and arrows are, respectively, marking examples of non-electroporated (GFP–ve) F4/80+ve and F4/80–ve mononucleated muscle cells. (B–D) CNTR–ve FACS plot referred to Figures 1I–K showing CD45+veCD31+ve cells (I), FAPs (J), and MuSCs (K) of TA of ∼4 months-old C57BL/6J mice injected with PBS and electroporated. [file Image_3.TIF]

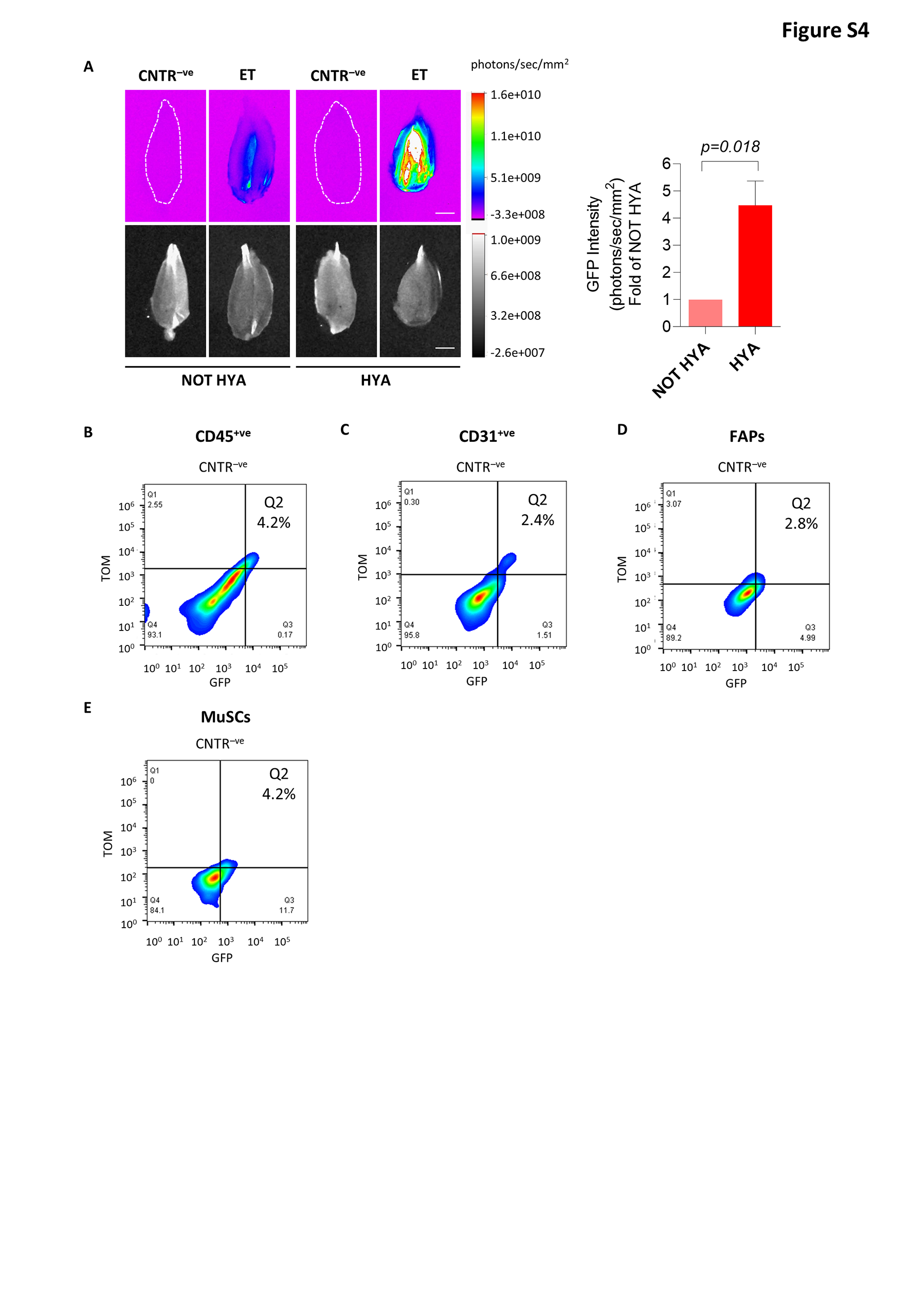

Supplement: Supplementary Figure 4 — Hyluronidase treatement in dystrophic muscles. (A) Representative Bruker In Vivo Xtreme I images (left) and GFP intensity measurement (photons/sec/mm2) (right) of dissected TA from ∼6 months-old mdx4Cv (MDX) mice injected with hyaluronidase (HYA) or not injected (NOT HYA). 2 h after hyaluronidase injection TA muscles were injected with PBS (CNTR–ve) or GFP plasmid (ET) and electroporated. Scale bar: 2.5 mm. White color in the figure (left, top panel) indicates photons/sec/mm2 > 1.6e+010. Corresponding reflectance images are shown in the bottom panel. Muscles were analyzed 1 week after electroporation. N = 3. (B–E) CNTR–ve FACS plots referred to Figures 4H–K showing CD45+ve cells (B), CD31+ve cells (C), FAPs (D), and MuSCs (E) of TA of ∼6 months-old mdx4Cv mice C57BL/6J mice injected with hyaluronidase, then injected with PBS and electroporated. [file Image_4.TIF]
